# Clinical application of improved VSD and VSD in the treatment of SSI after abdominal surgery: A retrospective randomized clinical study

**DOI:** 10.1097/MD.0000000000032785

**Published:** 2023-02-10

**Authors:** Tao Huang, Tong Liu, Mei Shang, Gang Han

**Affiliations:** a Department of Gastrointestinal Surgery, Wuhan Puren Hospital, Qingshan District, Wuhan, Hubei, China; b Department of Gastrointestinal Nutrition and Hernia Surgery, The Second Hospital of Jilin University, Nanguan District, Changchun, Jilin, China.

**Keywords:** abdominal surgery, improved vacuum sealing drainage, surgical site infection, vacuum sealing drainage

## Abstract

By comparing the efficacy and cost of improved vacuum-sealing drainage devices and vacuum-sealing drainage (VSD) devices in the treatment of postoperative abdominal surgical site infection, the clinical applicability and promotion of improved vacuum-sealing drainage devices were assessed. In our institution, between October 2019 and December 2021, 55 patients with surgical site infection after abdominal surgery were retrospectively analyzed, including 30 patients treated with improved VSD and 25 patients treated with VSD. The efficacy of wound healing, total dressing change cost throughout therapy, total hospital costs, hospital days, and bacterial culture results of wound secretions before and after treatment were compared between the 2 groups. Both groups achieved wound healing following vacuum sealing-drainage treatment, with no significant differences in wound healing time or secondary suture rate. However, the total hospitalization and dressing change costs in the improved VSD group were significantly lower than those in the VSD group. Similar to VSD, improved VSD is effective in the treatment of surgical site infections after abdominal surgery. Compared with VSD, the improved VSD device has lower dressing change costs and total hospitalization costs during the treatment process. The improved VSD has a wide range of applications and is suitable for clinical use and promotion.

**Key point**

- The drainage tube in the enhanced vacuum-sealing drainage device was a double casing pipe.
- Compared with the suction tube used in the simple negative pressure drainage device in previous studies, the drainage effect is better because of the unique structure of the double - casing pipe (Fig. 1).
- A sputum suction tube was used as the internal suction tube, and a soft and durable disposable anal canal was chosen as the exterior cannula.
- This design prevents the dressing from adhering to the tube wall of the internal suction tube, which would otherwise cause inadequate drainage and wound exudate infiltration between the inner and outer tubes.
- There is a gap of 0.3 to 0.6 cm, which is subsequently sucked out by an internal suction tube with negative pressure, and the number and size of the lateral aperture of the double casing pipe can be increased according to the drainage characteristics.

**Figure 1.**
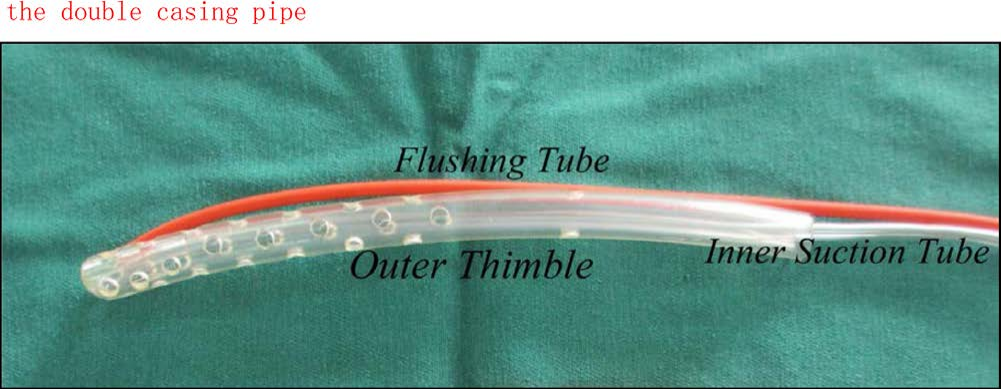
The double casing pipe.

## 1. Introduction

Surgical site infection (SSI) develops at the surgical incision and in the organ space after surgery. The most common postoperative complication is SSI,^[1]^ which includes superficial surgical site infection, deep surgical site infection, and organ space infection. According to the 2018 world health organization SSI prevention guidelines, the incidence of SSI in low-and middle-income countries ranges from 1.2% to 23.6%.^[2]^ Although the fact that the prevalence of SSI is significantly lower in high-income countries, it remains the second most prevalent health care associated infection in Europe and the United States (USA). The hospitalization costs of each SSI patient increased by $20,842 and their length of stay increases by 9.7 days.^[3]^ According to the NICE guideline, SSI patients’ hospitalization costs increase by nearly $90,000 per patient. SSI is associated with an increased risk of surgical morbidity, prolonged hospitalization, chemotherapy postponement, increased health care expenses, and worse long-term outcomes in some cases. Improved vacuum sealing drainage (VSD) offers numerous advantages in the treatment of surgical site infections, is a novel strategy for the treatment of surgical site infections, and represents a revolutionary advance.^[4]^ VSD can reduce patient pain, reduce staff responsibilities, and provide a solid foundation for subsequent therapy.^[5,6]^ Owing to its prohibitively high price, it is currently impossible for a large number of people to obtain VSD. In clinical practice, many patients require it, particularly those with open abdominal cavities and intestinal fistulas. Incision drainage is difficult to control, and VSD is an essential technique for resolving this issue. Based on the theory and mechanism of action of the VSD. An improved vacuum sealing drainage device (improved VSD) was built based on the theory and mechanism of action of the VSD. This study compared and analyzed the effectiveness and cost of vacuum-sealed drainage and improved vacuum-sealed drainage in the treatment of surgical site infection after abdominal surgery.

## 2. Material and techniques

### 2.1. General knowledge

**Figure 2.**
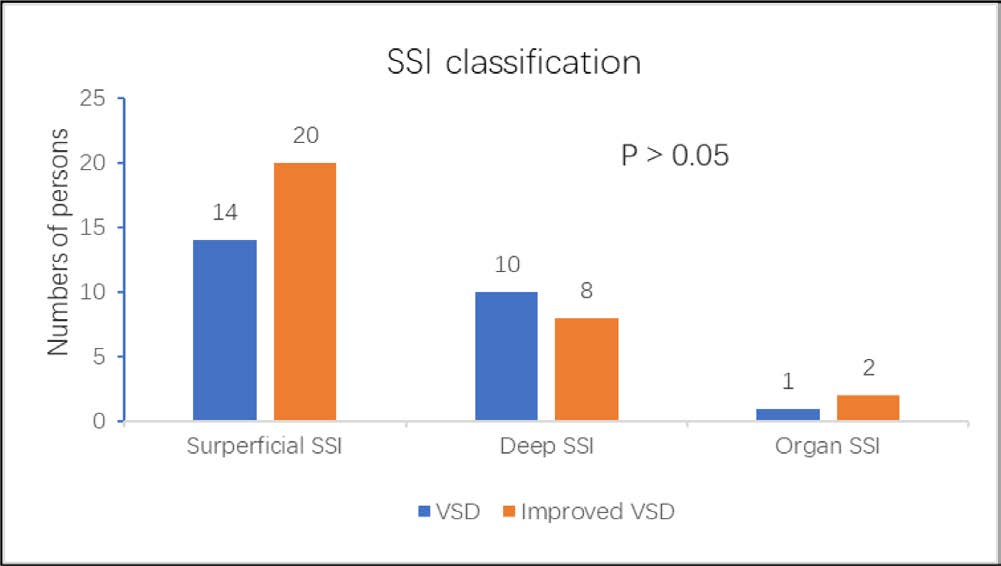
Number of subgroups in each SSI classification in the two groups. SSI = surgical site infection, VSD = improved vacuum-sealing drainage.

From October 2018 to October 2021, a retrospective study was conducted on 55 patients hospitalized with surgical site infection after abdominal surgery. Thirty patients with SSI were treated with improved vacuum drainage sealing. Twenty-five patients with SSI were treated with vacuum drainage. In the vacuum sealing drainage group, there were 13 males and 12 females ranging in age from 46 to 79 years, with a mean of (64.09 ± 15.07) years. There were 19 males and 11 females between the ages of 47 and 84 years in the improved vacuum-sealing drainage group, with a mean age of (52.16 ± 17.54) years. As shown in Tables 1 and 2, there were no statistically significant differences (*P* = .843) between the fundamental data of the 2 groups. As shown in Table 3, there was no statistically significant between the 2 groups in terms of the number of patients who underwent each type of surgery (*P* = .963). The number of patients classified as having surgical site infections did not differ significantly between the 2 groups (*P* = .224). (Fig. 2).

**Table 1:** Patient demographics.

Variable	VSD	Improved VSD	*P* value
Gender			
Male	13	19	.843
Female	11	11	
Age (yr)	64.09 ± 15.07	52.16 ± 17.54	.069
Re-operative hospital days (d)	2.27 ± 1.35	2.16 ± 0.96	.787
BMI (kg/m2)	23.74 ± 3.05	22.96 ± 4.07	.582
Pre-operative albumin (g/L)	120.36 ± 23.61	122.50 ± 29.41	.869
Incision length (cm)	8.73 ± 3.003	9.68 ± 4.61	.544
Preoperative blood glucose	7.37 ± 3.47	6.93 ± 2.34	.682
0peration time (min)	192.01 ± 80.81	182.84 ± 86.37	.777
NRS2002 Scores	2.36 ± 2.01	2.16 ± 1.91	.859
SSI occurrence time (d)	5.55 ± 2.02	5.74 ± 1.37	.759
DiabetesYes	3	5	.715
No	22	25	
Hypertension: Yes	1	4	.362
No	24	26	
Smoker: Yes	3	5	.715
No	22	25	

BMI = body mass index, NRS = nutritional risk screening, SSI = surgical site infection, VSD = improved vacuum sealing drainage.

**Table 2:** Patient demographics.

Variable	VSD	Improved VSD	*P* Value
Type of surgery			.963
Elective surgery	6	7	
Emergency surgery	19	23	
Surgical method			.838
Laparoscopic surgery	2	3	
Open surgery	16	21	
Laparoscopic-assisted	7	6	
Re-operation			.652
Yes	5	8	
No	20	22	
CDC classification			.244
Clean-contamina ted	13	22	
Contaminated	6	5	
Dirty-infected	6	3	
Prophylactic antibiotics			.521
Cefminoxine	12	19	
Ceftizoxime	7	6	
Etimicin	6	5	

VSD =improved vacuum-sealing drainage.

**Table 3:** Comparison of the number of different types of operations between the two groups.

Term of operation	VSD	Improved VSD	Χ^2^	*P* value
Radical resection of gastric cancer	22	33	2.76	.996
Radical resection for colon cancer	33	44		
Radical resection of sigmoid colon cancer	22	33		
Rectal Malignant Resection	33	55		
Open appendectomy	11	11		
Total proctocolectomy	22	11		
Exploratory laparotomy	55	77		
Intestinal fistula (laparotomy, adhesion lysis, intestinal resection, and anastomosis)	22	22		
Ileostomy	22	22		
Colostomy	33	22		

VSD = improved vacuum-sealing drainage.

### 2.2. Experimental materials and methods

#### 2.2..1. Experimental materials and equipment.

##### 2.2.1.1. VSD group.

1. Sterile black polyurethane foam dressings and polyvinyl-alcohol foam dressing software (Shenzhen Qi Kang Medical Instrument Co., LTD, China).
2. Bio occlusive transparent film (Manufacturer: Shenzhen Qi Kang Medical Equipment Co., Ltd).
3. Vacuum-flask device.
4. Central negative-pressure system.

##### 2.2.1.2. Improved VSD group.

1. Sterile degreased gauze.
2. Disposable enema packs.
3. Bio occlusive transparent film (Shenzhen Qi Kang Medical Instrument Co., Ltd.).
4. Suction catheter and gastric tube.
5. Bio occlusive transparent film
6. Central negative-pressure system.

#### 2.2.2. Treatment techniques.

When operating on patients with SSI, the aseptic principle must be adhered to scrupulously. Prior to routine debridement, bacterial culture and drug susceptibility testing of secretions should be conducted, and the wound surface should be washed repeatedly with hydrogen peroxide and 0.9% normal saline.^[7]^ Necrotic tissue, fascia, pus, and sutures were removed simultaneously removed from the affected area. Therefore, comprehensive debridement is necessary. It is conceivable that they would not be thoroughly cleaned in a single session. Multiple debridement procedures must be performed to ensure that no pus or necrotic material remains. Blood may seep from the granulation tissue during debridement. The bleeding must be halted. In addition, it is essential to evaluate the wound, assess its size and depth, and sneak it in the wound. The size and orientation of cavities must also be considered. If the incision contains an intestinal fistula, adequate drainage is performed.

### 2.3. VSD GROUP

Pending the outcome of the exploration incision, the PVA foam was cut and applied to the entire wound surface to ensure full contact with the wound surface. The outer layer of the biological semipermeable membrane is covered with foam. To achieve airtightness, the bio occlusive transparent film should reach 5 cm beyond the edge of the wound edge. When a drainage tube is difficult to seal, the “mesangial technique” should be employed to close it. The VSD device was sealed before use. The irrigation pipe and suction pipes were connected, and the irrigation pipe was filled with sterile saline solution. The suction pipe was connected to a negative- pressure system located at the center of the wall. The negative pressure was adjusted until the transparent layer outside the VSD device foam was compressed,^[8]^ and the foam was tightly associated with the wound surface, indicating that the negative pressure was adequate. Continuous normal saline irrigation was used to generate negative pressure drainage. The quantity of irrigation and the rate of dripping are determined by the state of the incision and characteristics of the drainage material.

### 2.4. Improved VSD group

**Figure 3.**
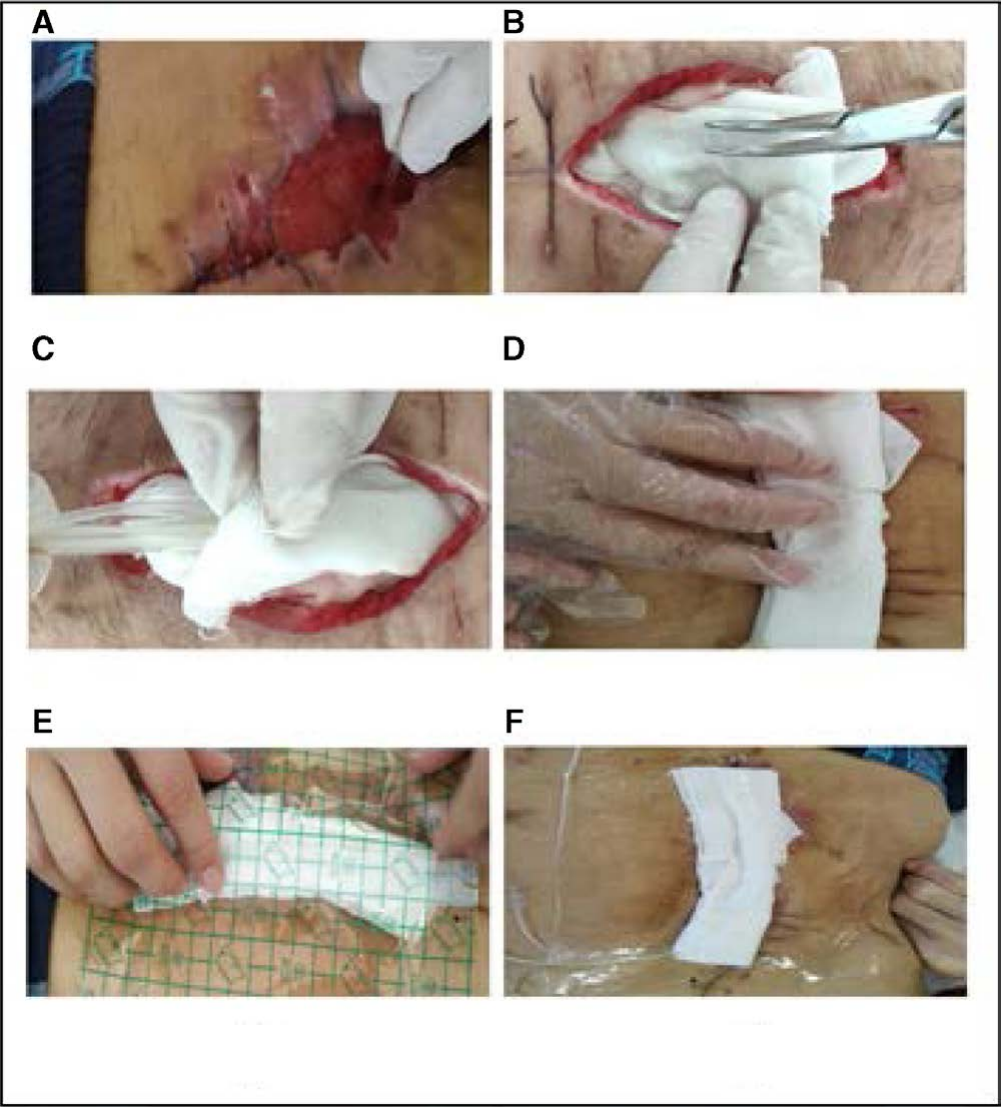
*Schematic diagram of operation Procedure. Note*. Installation renderings of Improved vacuum -sealing drainage device.

Step 1: Probe and rinse the incision. After wound debridement, it is necessary to fully explore the wound, observe the incision carefully, and measure the size and depth of the wound, and the size and direction of the cavities sneaking in the wound. After exploration, the wound was rinsed with flowing normal saline, as shown in Figure 3(A).

Step 2: The inner layer of the wound was filled with a gauze. Then, according to the results of wound exploration, sterile gauze was used to fill the wound and fill it fully. Attention should be paid to cavity sneaking in the wound to ensure that no dead space is left and to fill any gaps with gauze until it is level with the body surface, as shown in Figure 3(B).

Step 3: Placement of the double-casing pipe. The Li-style double casing pipe was laid flat onto the wound gauze. When the wound area is extensive or there are various cavities invade the wound, multiple the double-casing pipes can be used to increase drainage, and a 3-way tube can be used to connect the internal suction tubes, as shown in Figure 3 (C).

Step 4: Filling the outer layer of the wound with gauze: Place a layer of gauze outside the double-casing pipe. This construction resembles a hamburger, as shown in Figure 3(D), and is known as a Hamburg-type negative pressure drainage device.

Step 5: Closure: The next step in wound closure is to cover the wound with a film, To ensure the airtightness of the device, the surgical film should be roughly 4 to 5 cm larger than the edge of each wound before it is cut. Where a drainage tube is difficult to seal, the “mesangial technique” should be employed to close it, After the wound has been sealed, the centrifugal end of the suction tube is removed from the wound, connected to the connecting tube, and then to the negative pressure drainage bottle, as shown in Figure 3(E).

Step 6: The negative pressure device is connected to form a negative pressure device. Connect the suction pipe and the flushing pipe, connect the saline to the flushing pipe, connect the connecting pipe on the other connection port of the negative-pressure drainage bottle to the negative-pressure system in the middle of the hospital wall, and adjust the negative pressure. The outer transparent film of the VSD device covering the wound collapsed, and the dressing was tightly attached to the wound, suggesting that the negative pressure worked. When the negative pressure stabilized, negative pressure drainage and continuous irrigation with normal saline were performed. The quantity of flushing and dripping speed were established based on the relationship between the incision condition and the volume of purulent discharges evacuated, as shown in Figure 3(F).

### 2.5. Other treatments

Simultaneous treatment and control of core disorders in both groups should be prioritized during the therapeutic process. The dressing may be changed to a normal dressing if any incision is made, during the closed negative pressure drainage process; the incision granulation tissue is fresh, flat, and granular; the granulation does not have edema; and the wound secretion is negative in bacterial culture. Hypertonic saline gauze and moist compression can be used to minimize tissue edema near the incision base. If the granulation tissue remains flat and new throughout the treatment procedure and the incision is still deep, the wound should be sutured as soon as possible to accelerate healing.

### 2.6. Key points of operation during secondary suturing

A full-thickness suture incision and no tension suture, the suture needle should not be too deep, avoid suturing to the intestinal wall, needle distance of 1.5 cm, stitching margin of not less than 3 cm, on the edge of the incision 2 cm, not suture, abdominal cavity, or incisional drainage outflow, the VSD on the surface of the incision, and then continue to use negative pressure drainage for a few days.

### 2.7. Evaluation criteria

The secondary wound suture rate, wound healing duration, total dressing cost, total hospitalization cost, and wound exudation culture results before and after therapy were compared between the 2 groups.

### 2.8. Statistical analysis

Statistical analyses were performed using SPSS for MAC (, version 26.0; (IBM Corporation, Armonk, New York). The Categorical data, (e.g., gender, American society of anesthesiologists-classification, type of surgery, previous surgery, Surgical method, CDC classification, Prophylactic antibiotics, wound healing and term of operation) were compared using the chi-square test or Fishers exact test. Student *t* test was used to analyze normally distributed continuous variables, reported as means ± SDs, and the Mann–Whitney *U* test was used to compare abnormally distributed variables (e.g., re-operative hospital days, age, body mass index, operation time, bleeding volume during operation, wound healing time, length of stay, total dressing cost, and hospital expenses).

## 3. Results

There was no statistically significant difference in the secondary suture rates between the 2 groups (*P* = .392). As shown in Table 4, no patients experienced fatality, adverse reactions, wound bleeding, or other discomfort during the therapy procedure. Although a few patients developed minor blisters due to frequent film applications, the total exchanges and hospital expenses of the improved VSD group were significantly lower than those of the VSD group. This difference was statistically significant (*P* = .001), Table 5.

**Table 4:** Comparison of two sets of incision healing effects.

Clinical data	VSD	Improved VSD	*P* value
Wound healing:direct healing (n, %)	13 (.520)	19 (.633)	.392
Second phase suture (n,%)	12 (.480)	11 (.367)	

VSD = improved vacuum-sealing drainage.

**Table 5:** Comparison of clinical outcomes.

Clinical data	VSD	Improved VSD	*P* value
Healing time (d)	16.64 ± 1.64	19.58 ± 1.74	.269
total dressing cost (yuan)	16963.94 ± 1238.02	5058.04 ± 1875.97	.001
Hospital expenses (yuan)	109581.05 ± 18911.24	94581.05 ± 11541.76	.032

VSD = improved vacuum-sealing drainage.

### 3.1. Comparison of bacterial negative conversion rates

The wounds of both groups of patients were completely negative for bacterial culture by the third week of therapy, and there was no significant difference between the 2 groups in the first and second weeks (*P* = .748), as indicated in Table 6.

**Table 6:** The bacterial negative rate of the wound after one and two weeks of therapy.

GROUP	Positive bacteria culture before treatment	Negative bacteria culture after one week of treatment	Negative bacteria culture after two weeks of treatment
VSD	19	12 (.632)	17 (.895)
Improved VSD	24	14 (.583)	22 (.916)
*P* value		.748	.806

VSD = improved vacuum-sealing drainage.

## 4. Discussion

Many factors, including diabetes, hypoproteinemia, a high number of bacteria in the infected incision, necrotic fascia, and tissue edema,^[9–11]^ impede the healing of abdominal SSI; nonetheless, inadequate drainage remains the most significant problem. A VSD converts an open wound into a closed wound, preventing contamination and infection.^[12–14]^ The VSD is a point-like planar drainage that varies from the previous drainage, Continuous high negative pressure drainage from the VSD exerts a centripetal pulling force on the incision, which is superior to centrifugal compression produced by conventional gauze packing. The effects of NPWT include the fast removal of interstitial fluid through the negative pressure^[15]^ decrease in local edema, increased blood flow and therefore decrease tissue bacterial loads as well as decrease tissue edema.^[16,17]^ VSD improves wound microcirculation blood flow velocity, raises perfusion, accelerates necrotic substance absorption, increases the formation of granulation tissue,^[18]^ reduces bacterial burden,^[19–21]^ proinflammatory cytokines and proteases,^[22]^ and constricts hemostasis. These aspects work together to prevent incision infection, accelerate wound healing, minimize incision healing time, reduce the number of wound dressing changes, alleviate patient pain during dressing changes, and reduce patient stress. Currently, the cost of clinically available VSD is extremely high. In accordance with the theory and mechanism of action of the VSD, we developed an enhanced vacuum sealing drainage device (improved VSD) using inexpensive and readily available materials and shortened the dressing change. The interval is (1–2 days),^[23]^ although the current mainstream VSD demands a lengthy period between dressing changes (5–7 days), necrotic fascia or sutures, and pus moss in the wound should be removed on time.

Owning to the material device utilized, industrial VSD are relatively expensive. However, based on the central concept of industrial VSD, we constructed an improved vacuum sealing drainage device (improved VSD) using inexpensive, clinically available materials and reduced the period between dressing changes to every 3 days (1–2 days). However, the current mainstream closed drainage approach has a lengthy dressing change interval (5–7 days)^[24]^ and can remove necrotic fascia or sutures and pus moss in the incision at a later time, as well as detect the infection center that was not found during the initial debridement. Each time, the incision was cleaned with hydrogen peroxide and saline to reduce the number of germs. The comparison indicated that the wounds healed successfully in both the groups. At 1 week and 2 weeks after treatment, there was no statistical difference in the rate of secondary suture, the period of incision healing, or the rate of bacterial negative conversion between the 2 groups (*P* > .05), nor was there a statistical difference in the group with better VSD. The dressing change and total hospitalization costs were significantly lower than those in the VSD group (*P* < 0.05). We noticed that the improved VSD dressing utilized degreased gauze that stuck readily to the wound granulation tissue throughout the therapy. Before changing the dressing, rapid, continuous rinsing with 0.9% normal saline was performed for 10 minutes. When the dressing was replaced, the wound was infused with 0.9% normal saline. During the process of removing gauze removal, the dressing, specifically the interface between the wound granulation tissue and gauze, must be handled with care. After the aforementioned precautions were taken, the patient experienced no discomfort during the dressing change.

In clinical practice, for complex wounds, such as intestinal fistulas, particularly low intestinal fistulas and colon fistulas, the secretion is thicker mucus or feces, the diameter of the VSD drainage tube is modest, and the number of lateral apertures is fixed. Mucus and feces can obstruct the drainage tube, rendering it useless and delaying the healing time of the incision. This type of complex incision is frequently associated with abdominal wall abnormalities. The incision was large and contained several chambers. Compared to foam dressings, it is easier to pack a wound using gauze. In addition, the double-casing pipe can be inserted flexibly in accordance with the size and direction of the wound lacunae, and the number and size of the core of can be increased in accordance with the drainage characteristics. In addition, its short dressing cycle makes it easy to inspect the incision, locate infection foci missed during the initial debridement, and promptly remove dead tissue from the wound. Each time, the incision was cleansed with hydrogen peroxide and saline to minimize the quantity of bacteria in the incision and encourage wound healing. The dressing change interval was long in the VSD (5–7 days). If the initial wound debridement is insufficient or if a deep local infection is not identified, the replacement cycle will be lengthy, which will lengthen the incision healing period and increase the medical costs.

The present study has several limitations. First, this was a single-center retrospective randomized clinical study, and multicenter studies should be idealized. Second, the sample size of the present clinical trial was still small because there were few the patients with suffering SSI were few during the past 3 years at the Second Affiliated Hospital of Jilin University. The reasons may be various, but the primary reason may be the increasing popularity of laparoscopic surgery, which has the advantages of being less invasive, faster recovery, and less wound, but a sample size of 61 patients was also acceptable for this retrospective study. Finally, multicenter studies with a large sample size are required to further evaluate the efficacy of improved VSD in the treatment of SSI in the future.

## 5. Conclusion

VSD is a good wound treatment; however, its popularity and application are limited owning to its high price. The efficacy of the improved VSD in the treatment of SSI in this study was comparable to that of VSD, and its the price was only 1-third of its price. Because the selected materials are common and easy to obtain in the clinic, the price is low, the technique of application of VSD is easy to learn, and both nurses and residents can be replaced independently after training, which is conducive to its popularization and application.

## Acknowledgements

An Shang, Colorectal Section, Department of Surgery, The Second Hospital of Jilin University, No. 218, Ziqiang Dist, Changchun, 130041, Jilin.

## Author contributions

**Data curation:** Tong liu, Mei Shang.

**Formal analysis:** Tao Huang.

**Methodology:** Gang Han.

**Writing – original draft:** Tong liu.

**Writing – review & editing:** Tao Huang, Gang Han.
